# Development of a Phantom Limb Pain Model in Rats: Behavioral and Histochemical Evaluation

**DOI:** 10.3389/fpain.2021.675232

**Published:** 2021-06-21

**Authors:** Stanislava Jergova, Heidy Martinez, Melissa Hernandez, Benjamin Schachner, Suzanne Gross, Jacqueline Sagen

**Affiliations:** Miller School of Medicine, University of Miami, Miami, FL, United States

**Keywords:** phantom limb pain, axotomy, autotomy, Nav 1.7, GAD65/67

## Abstract

Therapeutic strategies targeting phantom limb pain (PLP) provide inadequate pain relief; therefore, a robust and clinically relevant animal model is necessary. Animal models of PLP are based on a deafferentation injury followed by autotomy behavior. Clinical studies have shown that the presence of pre-amputation pain increases the risk of developing PLP. In the current study, we used Sprague-Dawley male rats with formalin injections or constriction nerve injury at different sites or time points prior to axotomy to mimic clinical scenarios of pre-amputation inflammatory and neuropathic pain. Animals were scored daily for PLP autotomy behaviors, and several pain-related biomarkers were evaluated to discover possible underlying pathological changes. Majority displayed some degree of autotomy behavior following axotomy. Injury prior to axotomy led to more severe PLP behavior compared to animals without preceding injury. Autotomy behaviors were more directed toward the pretreatment insult origin, suggestive of pain memory. Increased levels of IL-1β in cerebrospinal fluid and enhanced microglial responses and the expression of NaV1.7 were observed in animals displaying more severe PLP outcomes. Decreased expression of GAD65/67 was consistent with greater PLP behavior. This study provides a preclinical basis for future understanding and treatment development in the management of PLP.

## Introduction

Amputations due to trauma or medical conditions often lead to the development of phantom sensations, perceived as they originate from the missing body part. In severe cases, phantom sensations can develop into a phantom pain. Phantom limb pain (PLP), a perception of pain from the missing extremities, is notoriously difficult to manage and can significantly impact the productivity and quality of life. The majority of amputees (50–85%) suffer from persistent or intermittent pain sensations associated with the limb that has been removed, which is often debilitating (reported as severe pain in over 30%), and interferes with daily activity, rehabilitation therapies, and utilization of prosthetic limb devices (1–5).

Despite the high prevalence and severity of PLP in amputees, mechanisms underlying PLP are poorly understood and current therapeutic options are mostly only marginally effective. Aberrant contributing peripheral and central neural processes that have gained consensus as proposed mechanisms include a likely progression from peripheral neuroma formation, to dorsal horn inflammation and disinhibition, and later cortical reorganization (6–12).

To be able to understand and properly treat PLP, the development of a replicable and valid animal model is necessary. Since animal models generally rely on an observable and measurable behavioral outcome, a significant roadblock in the development of a model for PLP is the inability to measure behavioral pain-related responses to stimuli post-amputation. Thus, an alternative strategy has been developed to mimic this condition. Animal models of PLP are based on deafferentation injury (complete nerve transection/neurectomy), producing self-directed autotomy behavior. Evidence has suggested that autotomy is a response to painful or dysesthetic sensations referred to as a denervated limb or region (13–18).

A key observation following medically necessary amputation is that the presence of pre-amputation pain is associated with the severity of subsequent PLP development in limb amputees, and can be predictive of its quality and location (19–25). This phenomenon is supported by preclinical findings in rats showing enhanced autotomy by a noxious insult prior to transection (15, 18) and by autotomy preferentially directed to the pre-injured site (20). However, little or no follow-up on this model development or utilization has been described in the subsequent literature, and progress in developing more effective therapeutics for the management of PLP has been slow.

Traumatic injuries to extremities necessitating medically indicated amputations often include prior peripheral nerve injury and inflammation in surrounding tissues that are too severe for a successful repair. Thus, the goal of this study was to develop and characterize a reproducible model of PLP to include components of these complex extremity injuries. The study also compared the onset and the severity of PLP-like behavior after different types or timing of the pre-amputation injury. A preliminary report has been presented previously (26).

## Materials and Methods

### Animals

Male Sprague-Dawley rats (~200 g, Envigo, IN) were housed in pairs with food and water *ad libitum* throughout the experiment, on corncob bedding (Envigo, IN) in 12-h light/dark cycle. Animals were observed daily for PLP scoring and for overall health status. Experimental procedures were reviewed and approved by the University of Miami Institutional Animal Care and Use Committee and the Department of Defense Animal Care and Use Review Office and followed the recommendations of the “Guide for the Care and Use of Laboratory Animals” (National Research Council).

### Study Design

Data from the preliminary experiments were used to design the current study. Using the methods described below, we have followed three different scenarios of pre-axotomy injury: Group A was injected with formalin (*n* = 9) or saline (*n* = 10) into lateral plantar hind paw followed by sciatic/saphenous axotomy 2 h later. Group B underwent injection of formalin (*n* = 9) or saline (*n* = 9) into medial plantar hind paw followed by axotomy 2 h later. Group C underwent chronic constriction injury (CCI) (*n* = 8/group) or sham injury (*n* = 8/group) of the sciatic nerve followed by axotomy at 1 day or 4 weeks later.

### Axotomy PLP Model

To model complete nerve severance in limb amputation, animals underwent unilateral sciatic, and saphenous transection surgeries. This was done at different time points following the pre-axotomy insults as described in the below sections. For all surgeries, aseptic surgical techniques were used. To perform the transection, animals were anesthetized by 4–5% isoflurane in O_2_ and maintained on 2% during surgery. The sciatic nerve was exposed at mid-thigh level, gently lifted, tightly ligated with silk proximal to trifurcation, and cut distally from the ligature. The saphenous nerve was exposed, tightly ligated with silk, and cut distally from the ligature (20). Muscle and skin were sutured, and rats were placed on a heated pad to recover. Control animals underwent sham procedures only.

### Formalin Injections

Intraplantar injection of formalin was used as a form of pre-amputation insult in order to produce extremity neurogenic inflammation as might be experienced following complex limb injuries that lead to medically indicated amputation. Rats were gently wrapped in a towel, and 5% formalin solution was slowly injected into the plantar region in a volume of 50 μl. An equal volume of saline was injected in the control group. The inflammatory injection was targeted to either the medial or lateral plantar hindpaw to determine the effect of pre-amputation pain location on subsequently directed post-amputation autotomy. Axotomy of the sciatic nerve followed 2 h later. During the period following formalin injection, pain behaviors (flinching) and hindpaw swelling were observed, indicating inflammatory pain.

### Peripheral Nerve Constriction Injury

To model the clinical scenario for medically indicated amputations after traumatic injuries involving the peripheral nerve, CCI (27) was used as a pre-axotomy injury, at either 1 day or 4 weeks prior to the complete nerve transection. To induce CCI, animals were anesthetized, and the right common sciatic nerve was exposed at the mid-thigh level using blunt dissection. Four 4-0 chromic gut ligatures spaced about 1 mm apart were tied around the sciatic nerve proximal to the trifurcation, loosely constricting the nerve such that circulation through epineural vasculature was not completely blocked. The musculature was sutured together, and the skin was closed with veterinarian grade cyanoacrylate (Nexaband). Animals were returned to their cages to recover and were observed daily. Sham animals underwent the same procedure omitting the nerve ligatures.

Animals in CCI groups were tested for the presence of tactile and cold hypersensitivity before the axotomy. Baseline values were recorded before the CCI surgery, and again at 24 h prior to the axotomy as follows: For the assessment of tactile responses, the threshold level to an innocuous mechanical stimulus was measured with calibrated von Frey hairs ranging from 0.4 to 15 g applied to the plantar skin of the hindpaw (28). The filaments were applied sequentially with increasing force. A brisk hind paw withdrawal together with at least one other supraspinal pain-related behavior, such as turning the head toward the stimulus, licking or shaking the paw, was considered as a positive response. The Dixon up-down method was used to calculate 50% paw withdrawal threshold. For the assessment of cold sensitivity, a non-noxious cooling stimulus was evaluated using the acetone test (29). 100 μl of acetone was dropped onto the lateral margin of the plantar hind paw from a blunted 22-ga needle attached to a syringe. Acetone was applied to the hind paw 5 times, with about 1–2 min between applications. The total number of positive responses (at least two of the following: paw withdrawal, head turning toward stimulus, paw licking, and shaking) out of five was converted to a percent response frequency.

### Phantom Limb Pain Scoring

Starting the day after axotomy, rats were examined daily and scored for both individual digits and overall severity using a scale based on preliminary data and developed in conjunction with the University of Miami Biostatistics Core. Individual digits were scored for signs of autotomy using the following weighted scale: 1 point for nail-biting (mild autotomy, category I), 3 points for injury to distal digits (moderate autotomy; category II), and 5 points for injury encroachment to proximal digits (severe autotomy; category III). The total scores were designed to capture and distinguish overall autotomy severity as follows: severity level I for nail biting only (maximum 5 points), severity level II for any distal digit injury (5 + (3 × number of digits in category II); maximum 20 points), and severity level III for any proximal digit injury (20 + (5 × number of digits in category III); maximum 45 points). Animals were euthanized when they exhibited severe autotomy with encroachment of any proximal digit (a score of 25 or above), in order to minimize distress, with the day of termination recorded. When PLP total autotomy scores were below the cutoff (<25 points), the study was terminated at the endpoint of 34 days. The final score for each animal at termination was retained through the end of the study for comparisons between treatment groups.

### Immunohistochemical and Biochemical Evaluations

Upon reaching the cutoff time point or at the end of the study, spinal CSF, and spinal cord tissues were collected for further analyses. Tissues used for comparisons were either from animals that reached the end of the experiment or from animals that developed severe behavior at the latest time points (when possible), in order to keep the post-axotomy timing consistent.

Animals were perfused with saline followed by 4% paraformaldehyde, and the spinal cord was dissected, postfixed overnight, and cryoprotected with 30% sucrose. Serial sections were cut on a cryostat at 40 μm, and every 4th section was collected either as slide-mounted or as free-floating. Standard immunostaining was done with Iba1 (Wako, 1:1,000), NaV1.7, (Abcam, 1:500), CGRP (Bioss, 1:500), and GAD65/67 (1:200, Sigma) primary antibodies, followed by fluorescent secondary antibody (Alexa Fluor, 1:250). Controls were run with the omission of primary antibody.

Integrated density of immunostaining was evaluated by ImageJ. 20x images were transformed into grayscale with correction for the background. The area with a positive signal from Iba-1, CGRP, or GAD65/67 was outlined within the dorsal horn laminae I-III, and the density per area was measured. Eight to 10 serial lumbar spinal sections were evaluated per animal. For NaV immunostaining, the total length of the NaV-positive fibers in the lumbar dorsal horn was analyzed by Stereoinvestigator and the Spaceballs tool (MBF Bioscience). Eight to 10 serial sections from the lumbar spinal cord per animal were used for the analysis.

To perform ELISA for the detection of IL-1β, animals were euthanized by CO_2_; cerebrospinal fluid was collected *via* the aspiration from cisterna magna and stored at −80°C. Protein concentration in the CSF was determined by BCA (Thermo Scientific), and the samples were processed according to the manufacturer's protocol (Abcam, IL-1β kit).

### Statistical Analysis

SigmaStat software was used to estimate the sample size based on our preliminary data and experience with the CCI and formalin models, with *n* = 8–10 animals per group. GraphPad RandomNumbers calculator was used to randomly assign animals for experimental groups and to generate the order for surgical interventions. Animals were observed and scored daily for PLP behavior by experimenters blinded to pre-injury treatment. In CCI/sham pre-axotomy groups, animals were tested prior to the CCI injury and prior to axotomy (1 day or 4 weeks post-CCI/sham) by an experimenter blinded to surgical/treatment group. In the cases of animals that reached the cutoff point (PLP score over 25 points) before the end of the experiment, their last PLP score was used in the data analysis for the following time points through the end of the experiment.

PLP score data were analyzed by SigmaStat with the two-way RM ANOVA with Bonferroni post-test as data skewness was within the appropriate range for ANOVA. Student's *t*-test was used for comparison of two groups in autotomy onset/cutoff analysis and digit targeting, and the chi-square test for the analysis of percent remaining (after attrition due to reaching cutoff).

All samples for immunocytochemical and neurochemical evaluations were also analyzed in a blinded fashion. Immunohistochemical and biochemical data were evaluated using the *t*-test.

Correlation analysis of PLP data and histochemical and behavioral outcomes was performed using GraphPad single linear regression. *P* and R^2^ values are indicated.

*P*-values <0.05 were considered significant for all tests.

## Results

### Lateral Injection of Formalin

**Figure 1:**
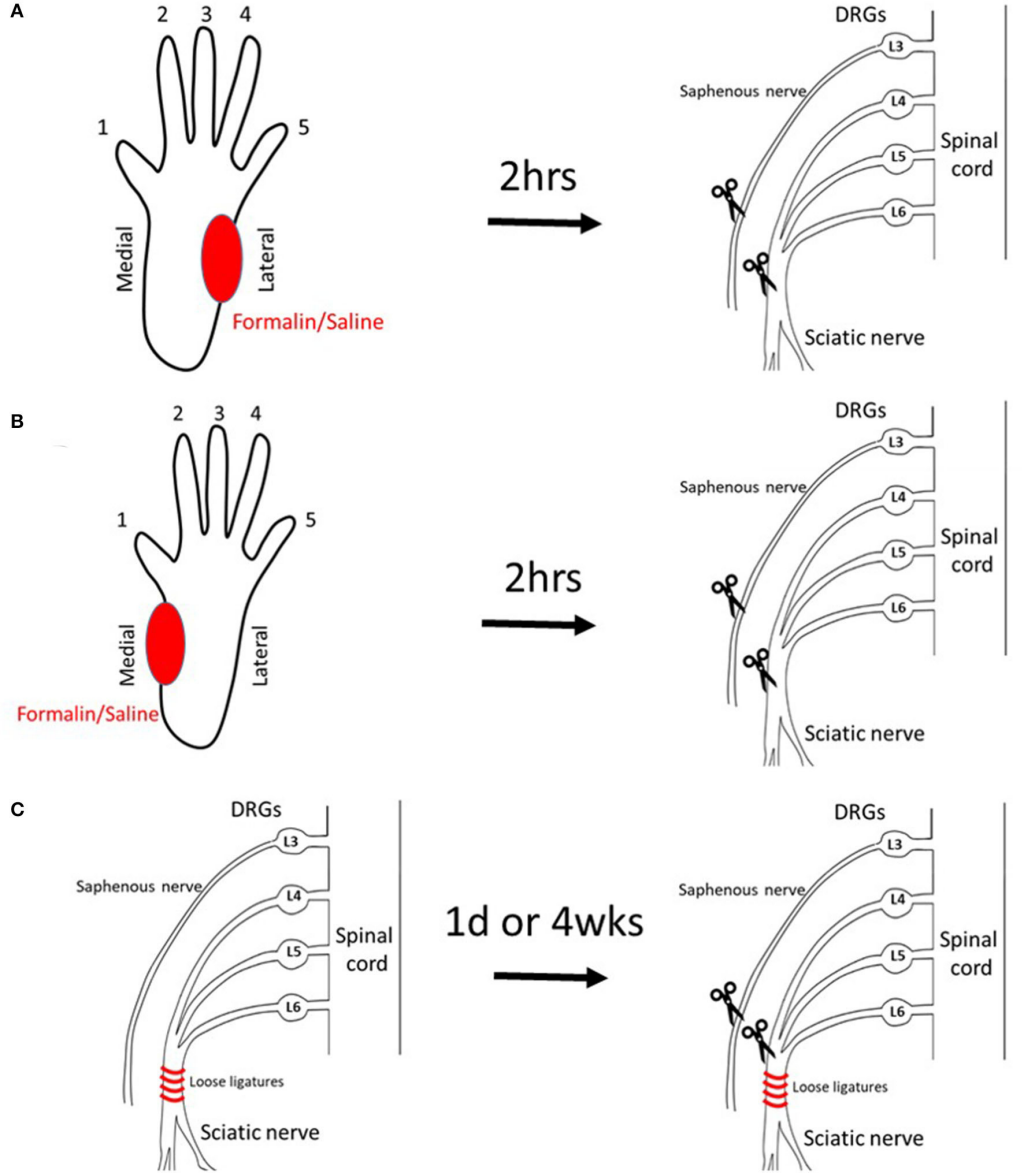
Models to induce PLP-like behavior in rats. **(A)** Injection of saline/formalin into the lateral plantar hindpaw 2 h prior to axotomy (lateral injection). **(B)** Injection of saline/formalin into the medial plantar hindpaw 2 h prior to axotomy (medial injection). **(C)** CCI at 1 day or 4 weeks prior to axotomy (CCI).

Five percent formalin (*n* = 9) or saline (*n* = 10) was injected into the lateral hind paw of naïve, healthy animals followed by complete transection of the sciatic and saphenous nerves 2 h later (Figure 1A diagram).

**Figure 2:**
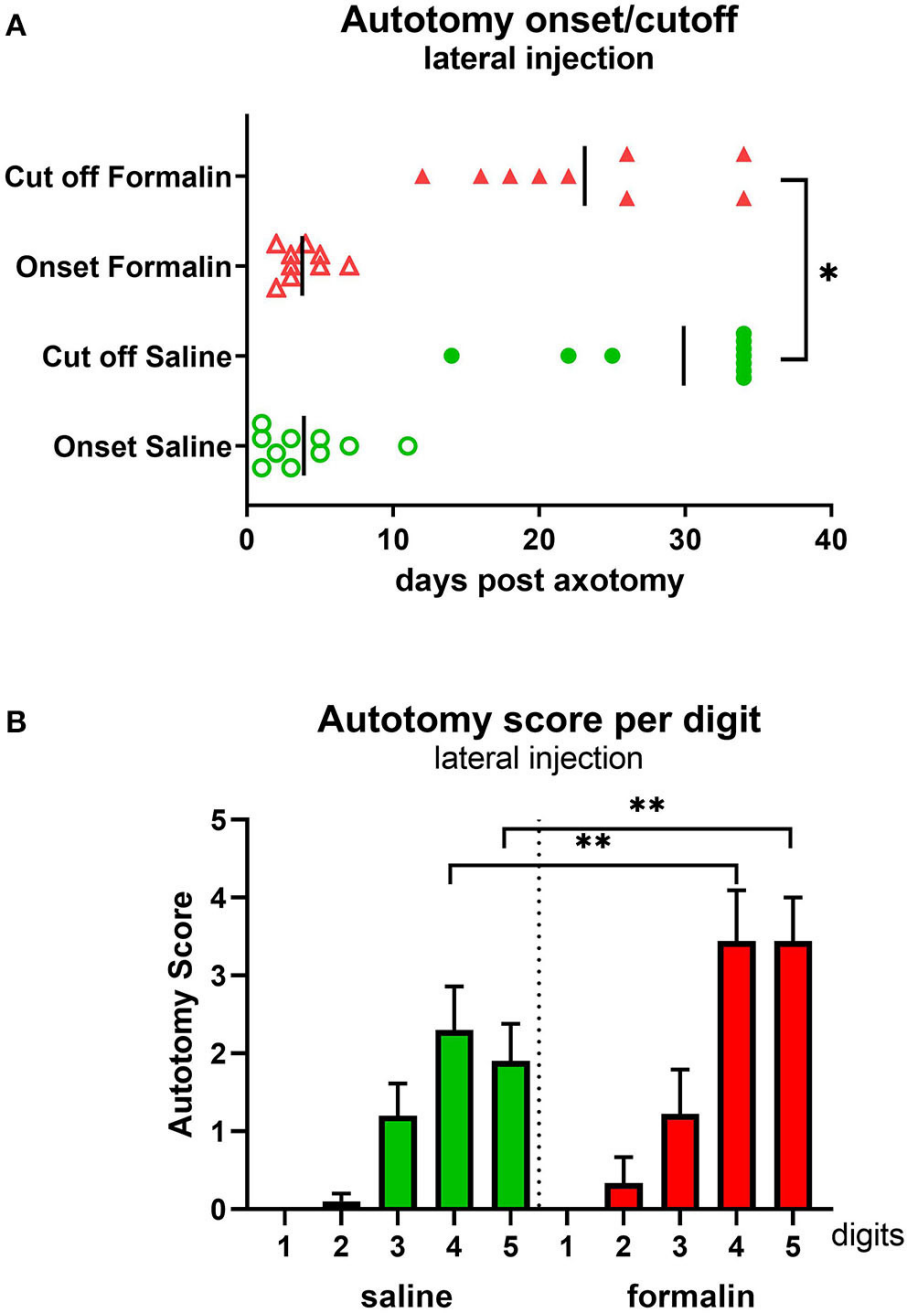
Autotomy onset and progression **(A)** and autotomy scores of individual digits **(B)** after lateral injection prior to axotomy. *P < 0.05, **P < 0.01 between saline- and formalin-pretreated groups. Vertical marks indicate the mean onset and cutoff times in each group.

Animals were observed and scored daily post-axotomy. The first signs of autotomy (biting the nails or minor injury to the tip of the toe) developed within a week post-axotomy in both groups (Figure 2A). The cutoff time point, either due to the severity of behavior when PLP score reached the threshold level (a score of 25 points or above) or due to planned termination of the study (day 34), was shifted to the left in the formalin group compared with the saline group (*p* < 0.05), indicating more animals in the formalin group being prematurely euthanized due to the severity of the behavior. Analysis of the affected digits at the time of perfusion showed higher scores (more severe injury) directed toward the lateral digits 4 and 5, paralleling the location of the pre-axotomy insult (*P* < 0.05 between digits 4.5 and 2.3). Higher severity scores directed toward those lateral digits were observed in the formalin group (*P* < 0.01 between formalin- and saline-injected groups for digits 4 and 5; Figure 2B).

**Figure 3:**
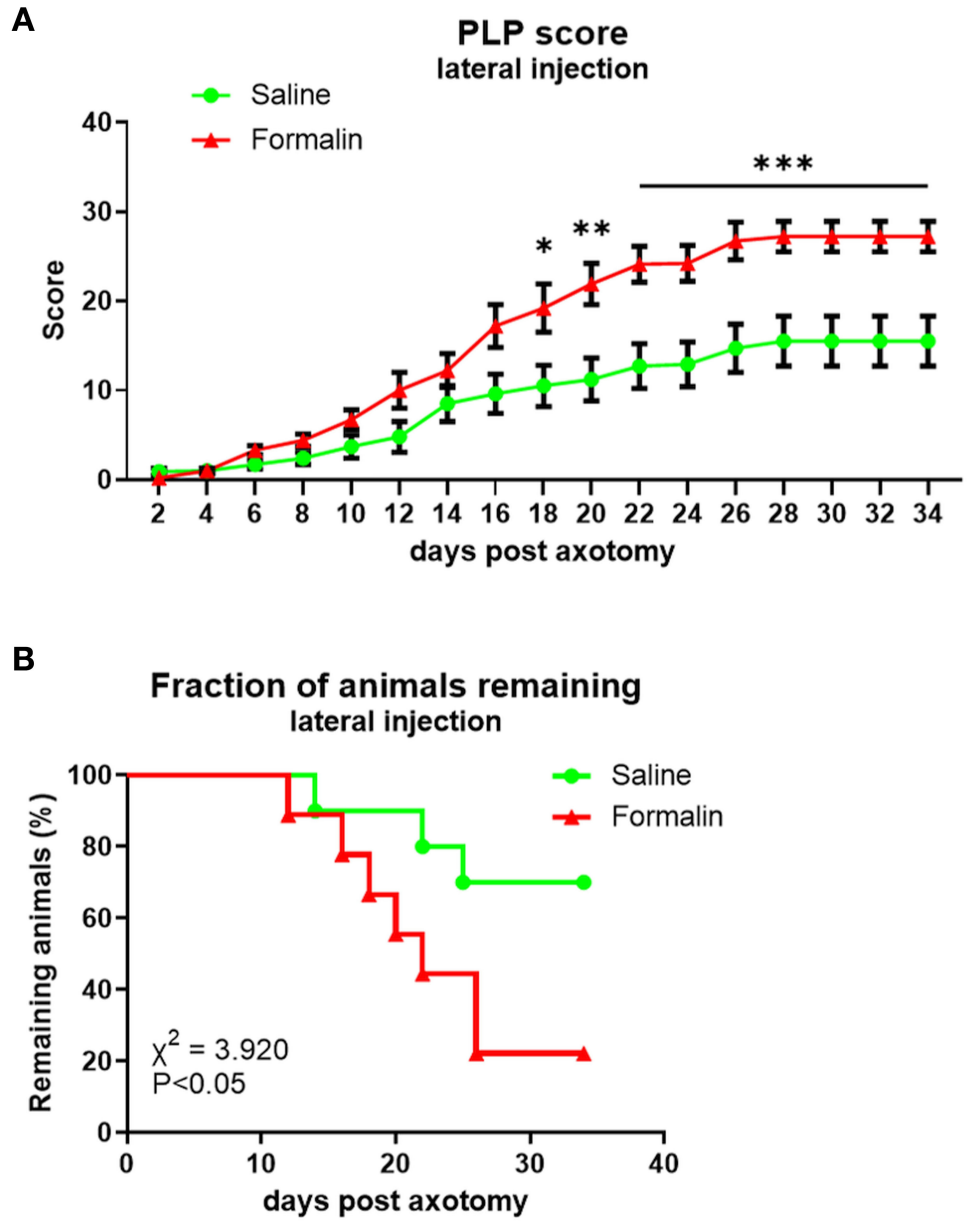
Time course of PLP development as indicated by autotomy scores **(A)** and fraction of animals remaining **(B)** over time after lateral injection prior to axotomy. *P < 0.05, **P < 0.01, ***P < 0.001 between saline- and formalin-pretreated groups.

PLP scores were significantly higher in the formalin group starting at ~3 weeks post-axotomy (Figure 3A) with overall *F*(dF_1, 33_) = 8.882, *P* < 0.001. This was also reflected in the declining percent of remaining animals in the formalin-treated group compared to saline-treated group due to more rapidly reaching cutoff autotomy severity (*P* < 0.05, Figure 3B). The severity of this behavior in the formalin-injected group started to increase more rapidly at ~2 weeks post-axotomy compared to the saline group, with most of the formalin-treated animals necessitating termination within 25 days post-axotomy. At the end of the experiment, the fraction of remaining animals was 22% of the formalin-injected animals and 70% of saline-injected animals.

### Medial Injection of Formalin

**Figure 4:**
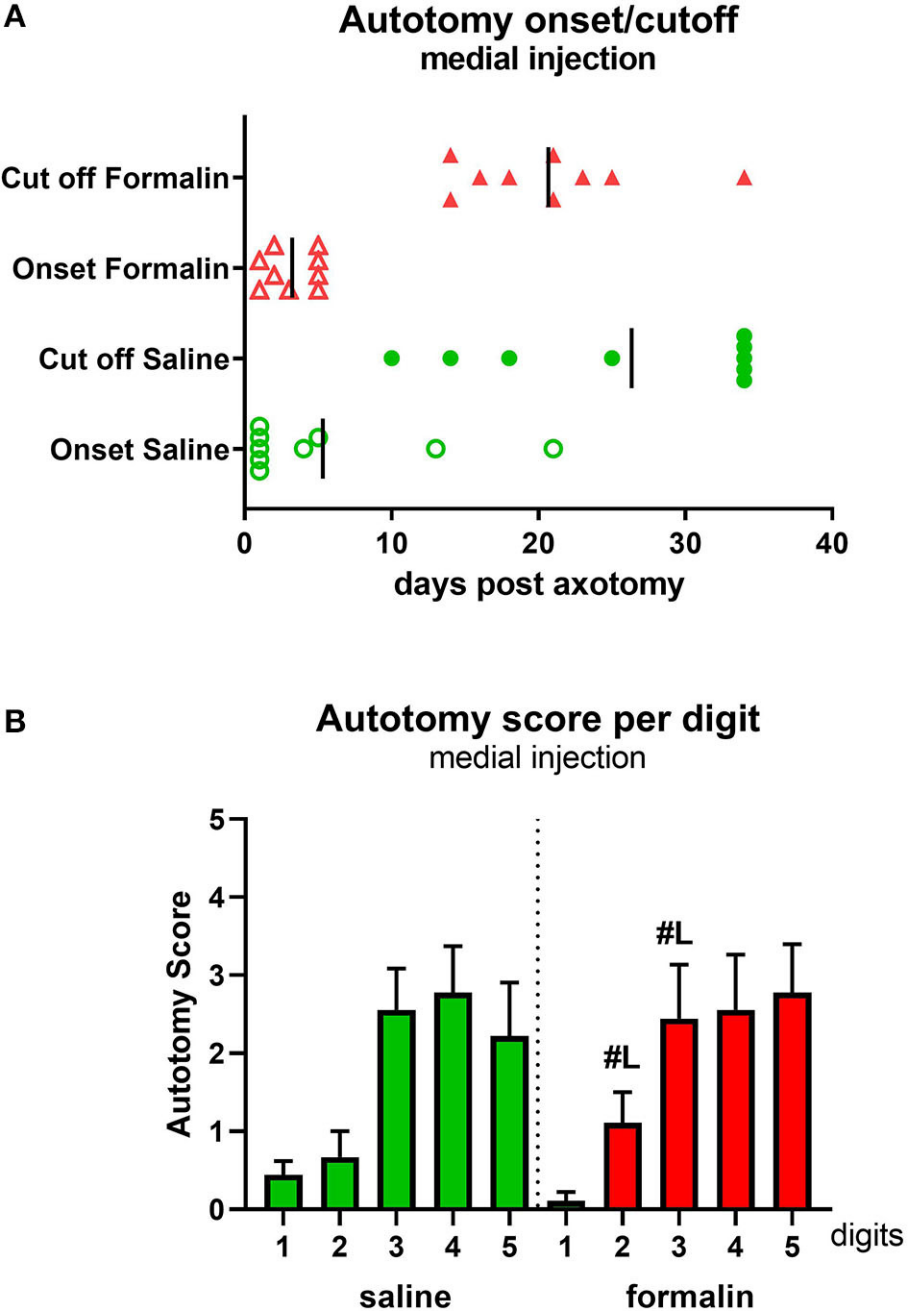
Autotomy onset and progression **(A)** and autotomy scores of individual digits **(B)** after medial injection prior to axotomy. Vertical marks indicate the mean onset and cutoff times in each group. ^#L^*p* < 0.05 vs. respective digits in lateral injection group.

Injection of saline (*n* = 9)/formalin (*n* = 9) into the medial hind paw if naïve, healthy animals, followed by axotomy 2 h later (Figure 1B diagram), resulted in similar behavioral progression as in lateral injections groups. The onset of the autotomy was observed within the first week post-injection, observed slightly earlier in the formalin group compared with saline, but this did not reach statistical significance (Figure 4A). Similarly, the cutoff time points were also reached earlier in the formalin group, although, in this medial injection case, differences between formalin and saline groups were not significant (*P* > 0.05).

Analysis of the affected hind paw digits showed a more broadly distributed severe behavior scoring compared to the lateral injection, with digits 3–5 targeted with moderate-high severity (Figure 4B), in contrast to the more laterally concentrated autotomy (digits 4 and 5) observed after the lateral formalin injection (Figure 2B). Comparisons were also made between individual digits in lateral vs. medial treated formalin groups. This revealed significantly higher autotomy scores in digits 2 and 3 following medial formalin injections compared with lateral formalin (*P* < 0.05, indicated on Figure 4B).

**Figure 5:**
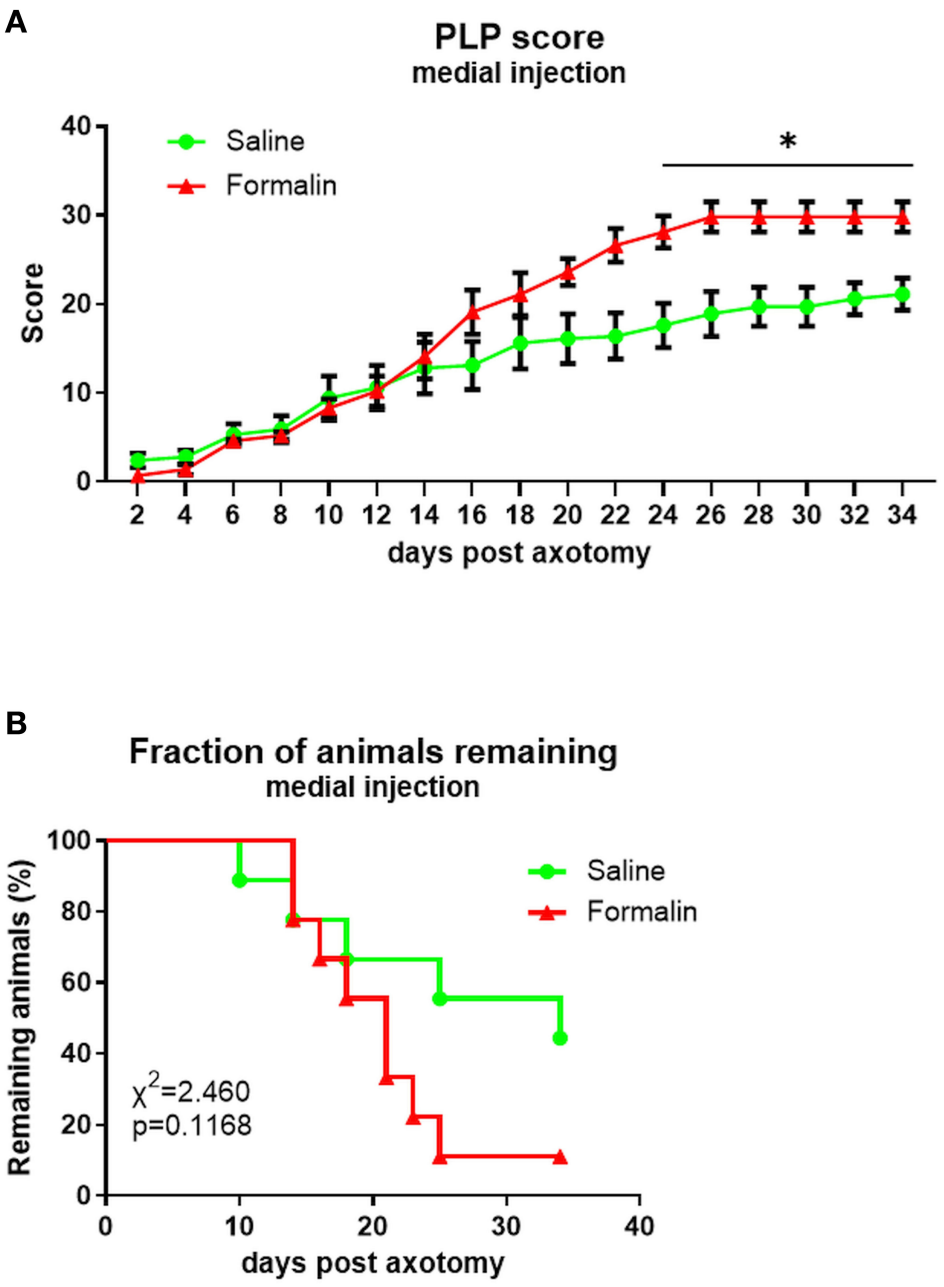
Time course of PLP development as indicated by autotomy scores **(A)** and fraction of animals remaining **(B)** over time after medial injection prior to axotomy. *P < 0.05 between saline- and formalin-pretreated groups.

The overall PLP score in both medially injected groups was comparable to those observed previously following lateral injections (Figure 5A). Differences in PLP severity scores between formalin-injected and saline-injected animals were observed starting at ~3 weeks post-axotomy, with overall *F*(dF_1, 33_) = 7.164, *P* < 0.001.

The attrition rate was higher in the medial formalin-injected group compared to saline, but even in the medial saline group, more than 50% of animals were terminated before the end of the experiment due to reaching cutoff severity. At the end of the experiment, the fraction of remaining animals was 11% of formalin-injected animals and 44% of saline-injected animals. The overall percent remaining was not significantly different between groups (*P* > 0.05, Figure 5B).

### Chronic Constriction Injury

To mimic the clinical scenario of a traumatic peripheral nerve preceding a medically necessitated amputation, animals underwent constriction of the sciatic nerve (or sham injury without the nerve constriction) followed by complete nerve transection either 1 day (CCI 1d) or 4 weeks (CCI 4w) after CCI (Figure 1C diagram, *n* = 8/group). Prior to the axotomy, animals were tested for the presence of hypersensitivity to tactile and cold stimuli (Supplementary Figure 1). Animals in both CCI groups developed hypersensitivity; no differences between pre- and post-injury were observed in sham groups.

**Figure 6:**
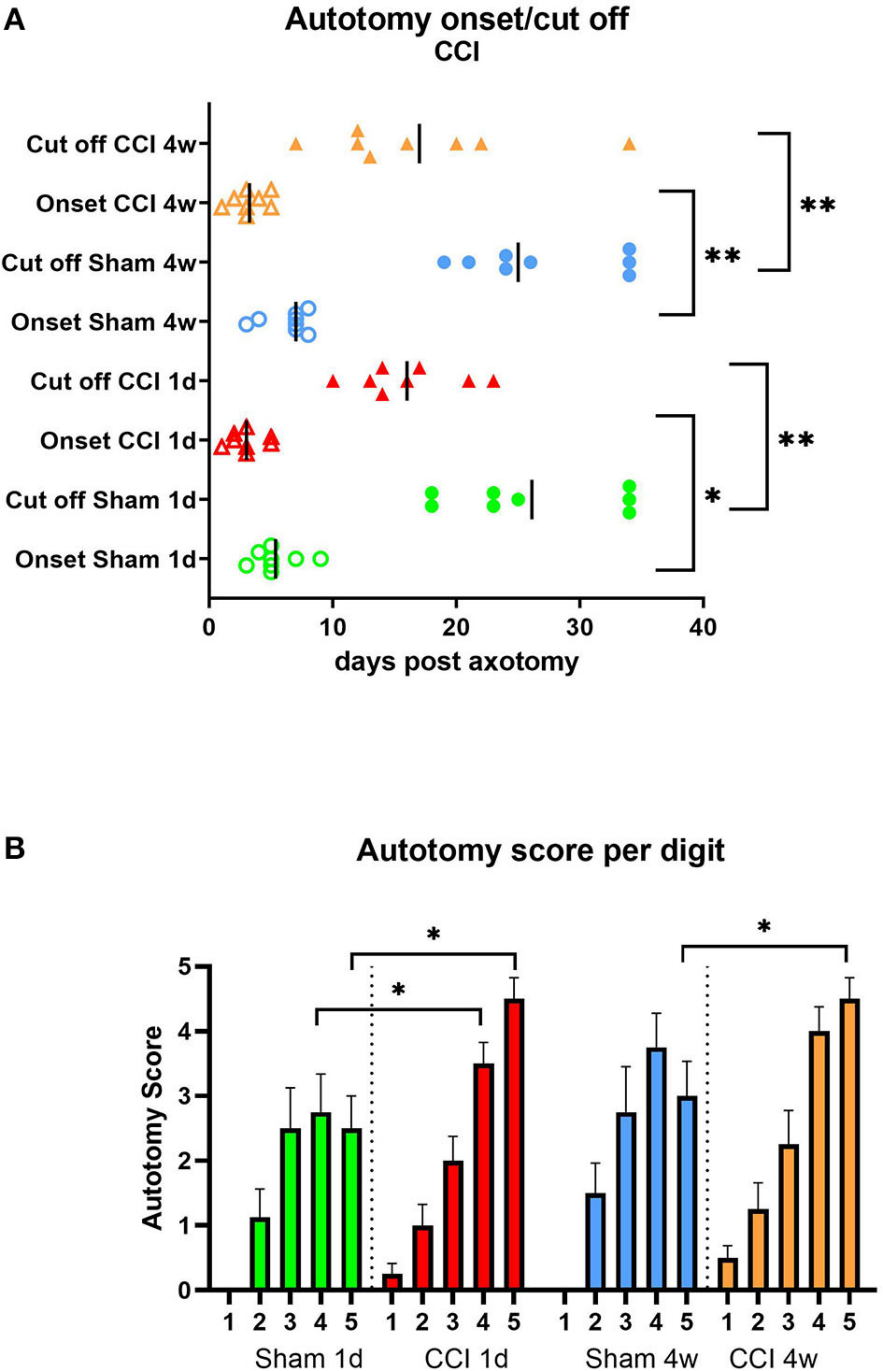
Autotomy onset and progression **(A)** and autotomy score of digits **(B)** after CCI prior axotomy. Vertical marks indicate the mean onset and cutoff times in each group. *P < 0.05, **P < 0.01 between indicated groups.

The onset of the autotomy behavior in all groups was within the first week post-axotomy, with the earliest signs observed in the CCI groups compared to sham group onset (*P* < 0.05 for CCI 1d compared to sham 1d and *P* < 0.01 for CCI 4w compared to sham 4w, Figure 6A). Similarly, the cutoff time point was reached earlier in CCI groups compared to the sham groups (*P* < 0.01 for both CCI groups compared to shams, respectively, Figure 6A). The analysis of the targeted digits showed differences in the autotomy scores between CCI and sham animals (*P* < 0.05 CCI vs. sham, digits 5 and 4, Figure 6B).

In addition, more severe autotomy behavior overall, and notably directed toward the lateral digits, was observed in the CCI-pretreated groups.

**Figure 7:**
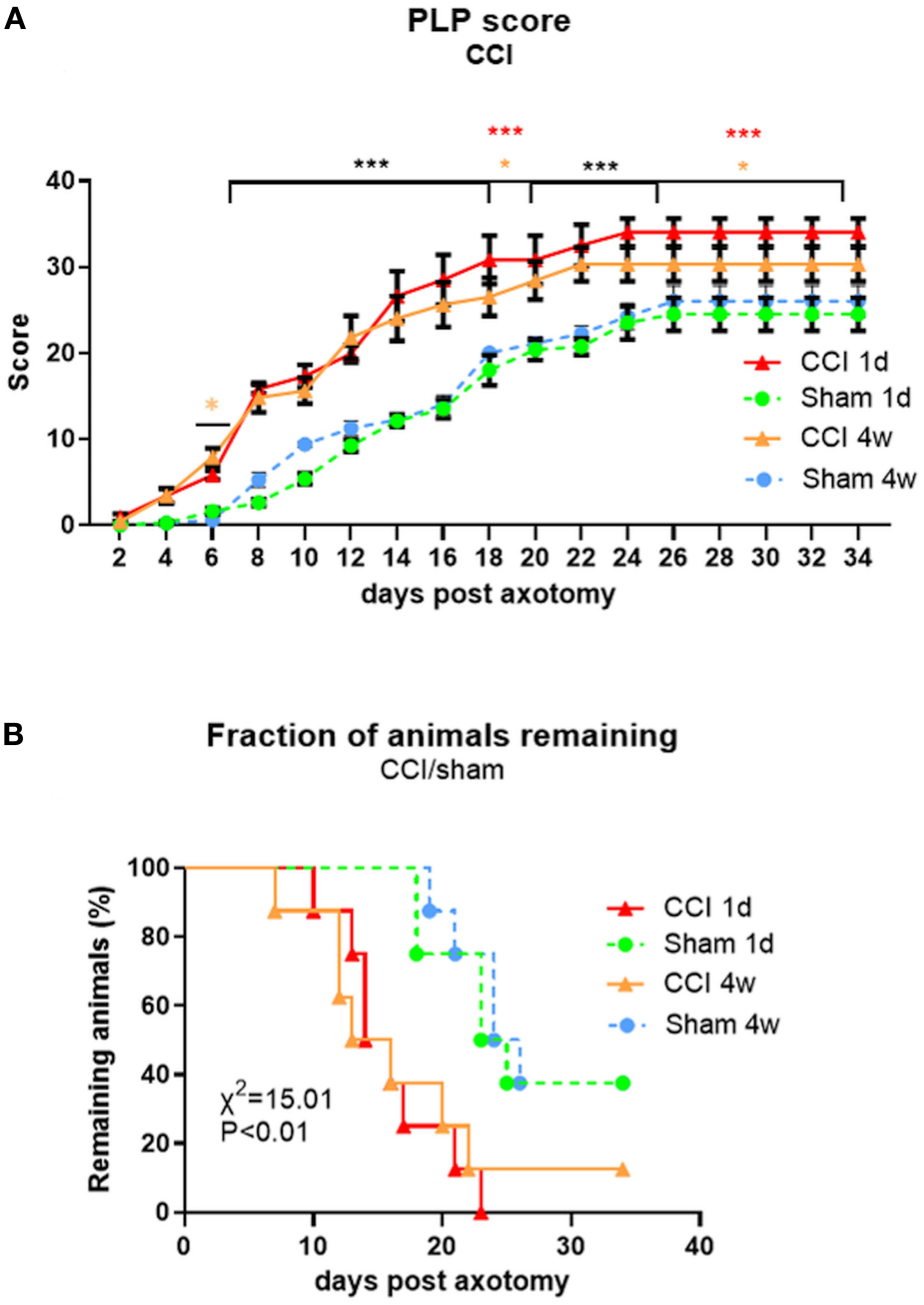
Time course of PLP development as indicated by autotomy scores **(A)** and fraction of animals remaining **(B)** over time after CCI (at 1 day or 4 weeks) prior to axotomy. *P < 0.05 for CCI 4w vs. Sham 4w (orange), ***P < 0.001 for both CCI vs. Sham (black) and CCI 1d vs. Sham 1d (red).

The overall PLP scores between sham and CCI groups were significantly different [*F*(dF3, 33) = 3.019, *p* < 0.001], starting within the first week post-axotomy (Figure 7A). The progress of PLP behavior was comparable between the two CCI groups with the earliest signs of severe behavior observed around day 10 post-axotomy. Significant differences between CCI and sham groups were observed starting by 6 days post-axotomy (*P* < 0.05 for CCI 4w vs. sham 4w), and continued with both CCI groups having significantly higher PLP scores compared to sham groups (*P* < 0.001 for days 7–18 for both post-CCI time points; *P* < 0.05 for CCI 4w and *P* < 0.001 for CCI 1d compared with the respective sham groups on days 18–20; *P* < 0.001 for both CCI groups compared to their respective shams up to day 25; and *P* < 0.001 for CCI 1d and *P* < 0.05 for CCI 4w from days 25–34). The fraction of remaining animals was lower in CCI groups (0% for CCI 1d and 12% for CCI 4w) compared to sham groups (almost 40%) due to the severity of the PLP behavior, with more rapid progression in CCI groups (*P* < 0.01 compared to sham groups, Figure 7B). Although, early axotomy (1 day post-CCI) appeared to produce more severe PLP scores than delayed axotomy (4 weeks post-CCI), the differences were not statistically significant.

**Figure 8:**
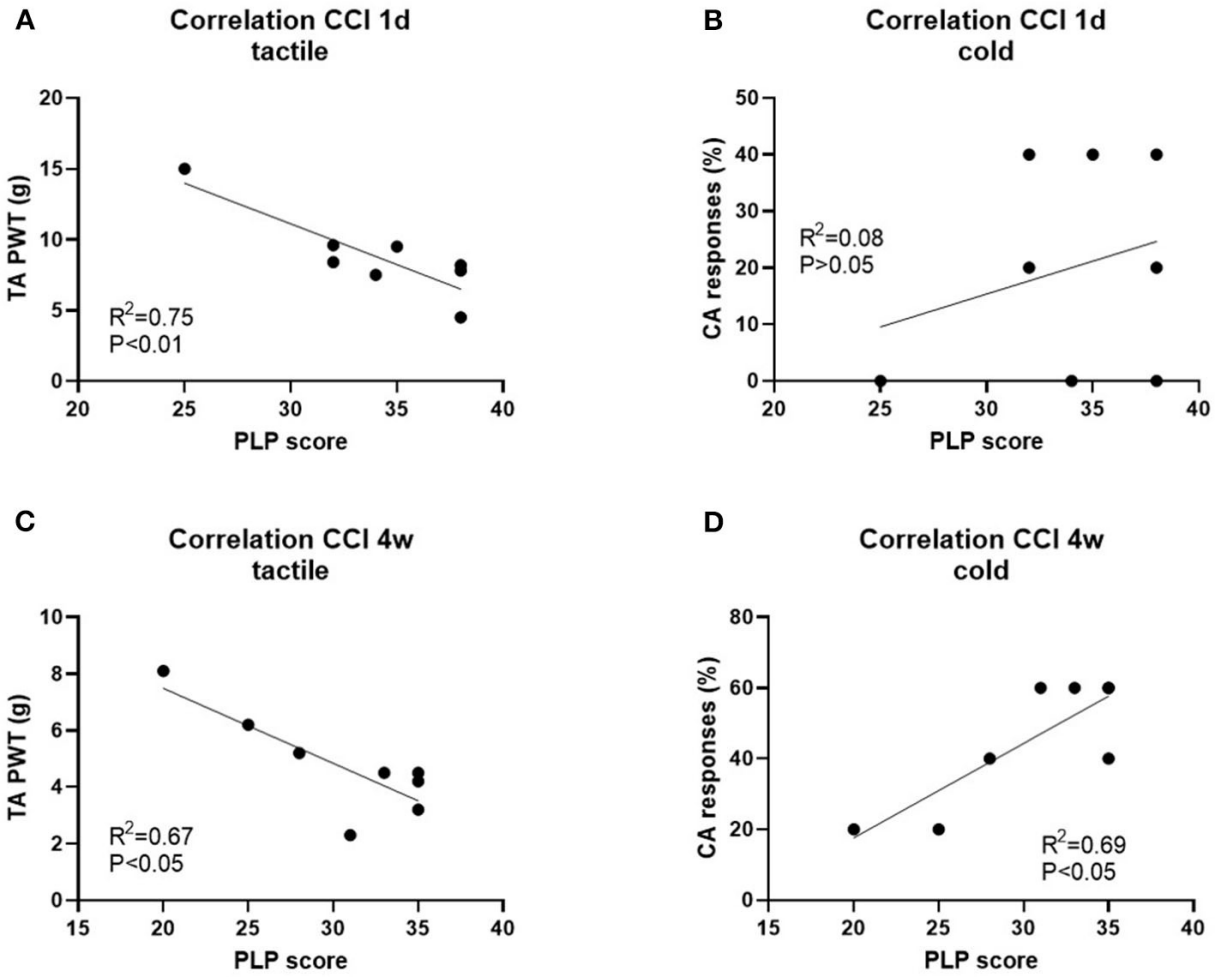
Correlation analysis of PLP scores post-axotomy and tactile hypersensitivity (tactile) and cold hypersensitivity (cold) after 1d **(A,B)** and 4 weeks **(C,D)** post-CCI, prior axotomy. R^2^ and *P*-values for each analysis are indicated.

Correlation analysis showed a relationship between tactile and cold hypersensitivity score before axotomy and the severity of PLP behavior after axotomy (Figure 8; P and R^2^ values are indicated).

### Immunochemical Analysis

Several pain-related biomarkers were evaluated in animals with either severe (>25 points) or mild (<25 points) PLP behavior to discover possible changes in the level of pain biomarkers in relation to the PLP severity. Only a subset of animals from each pretreatment was selected for this analysis, in order to compare changes in markers in non-overlapping PLP severity grouping. For these comparisons, animals with formalin, or saline pretreatment were used due to the limited number of animals with CCI injury with mild behavior. Correlation analysis of PLP scores and histochemical data were performed as well.

#### NaV 1.7

**Figure 9:**
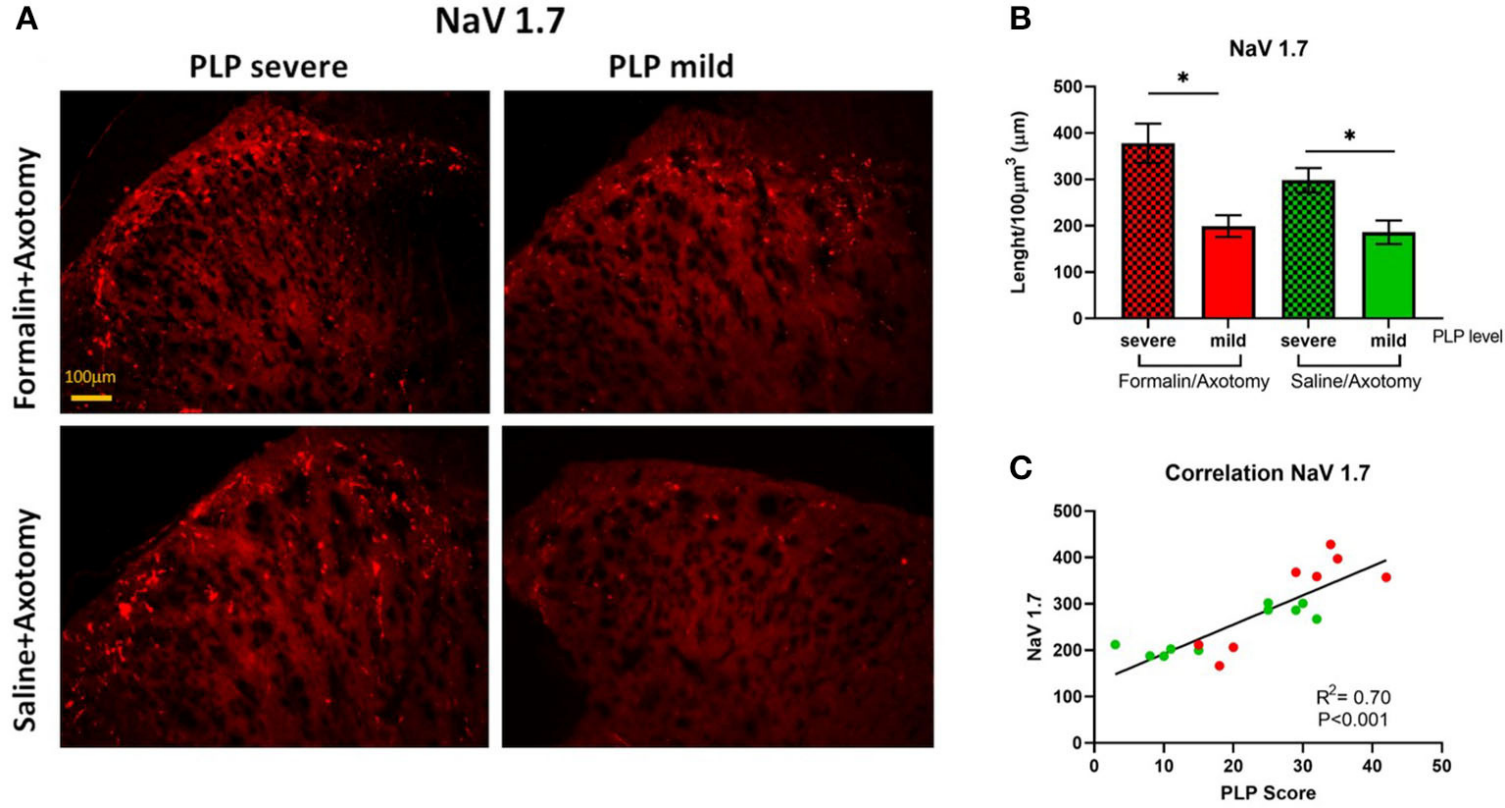
**(A)** Immunostaining of spinal dorsal horn for NaV1.7 in animals with severe and mild PLP behavior. **(B)** Evaluation of NaV1.7 total length in spinal cord from lumbar levels. **(C)** Correlation analysis between NaV 1.7 data and PLP scores. *P < 0.05 between the indicated groups.

The expression of sodium ion channels Nav1.7 was evaluated in the spinal dorsal horn at lumbar levels by measuring the total length of positive fibers. Significant changes were observed for NaV 1.7, showing upregulation in animals with severe PLP behaviors compared with those showing milder PLP (Figures 9A,B; *P* < 0.05). This upregulation was observed in both formalin- and saline-treated animals that showed higher PLP scores. The formalin-treated animals with high PLP scores appeared to show stronger NaV1.7 upregulation than the saline cohorts, but this did not reach statistical significance, likely due to the selection of the animals in both groups exhibiting high PLP scores for this comparison. Immunohistochemical staining showed positive fibers concentrated in the superficial laminae, extending to lamina III (Figure 9A). Correlation analysis showed a positive correlation between the length of NaV 1.7 fibers and the PLP scores (*P* < 0.001, Figure 9C).

#### Iba-1 and IL-1β

**Figure 10:**
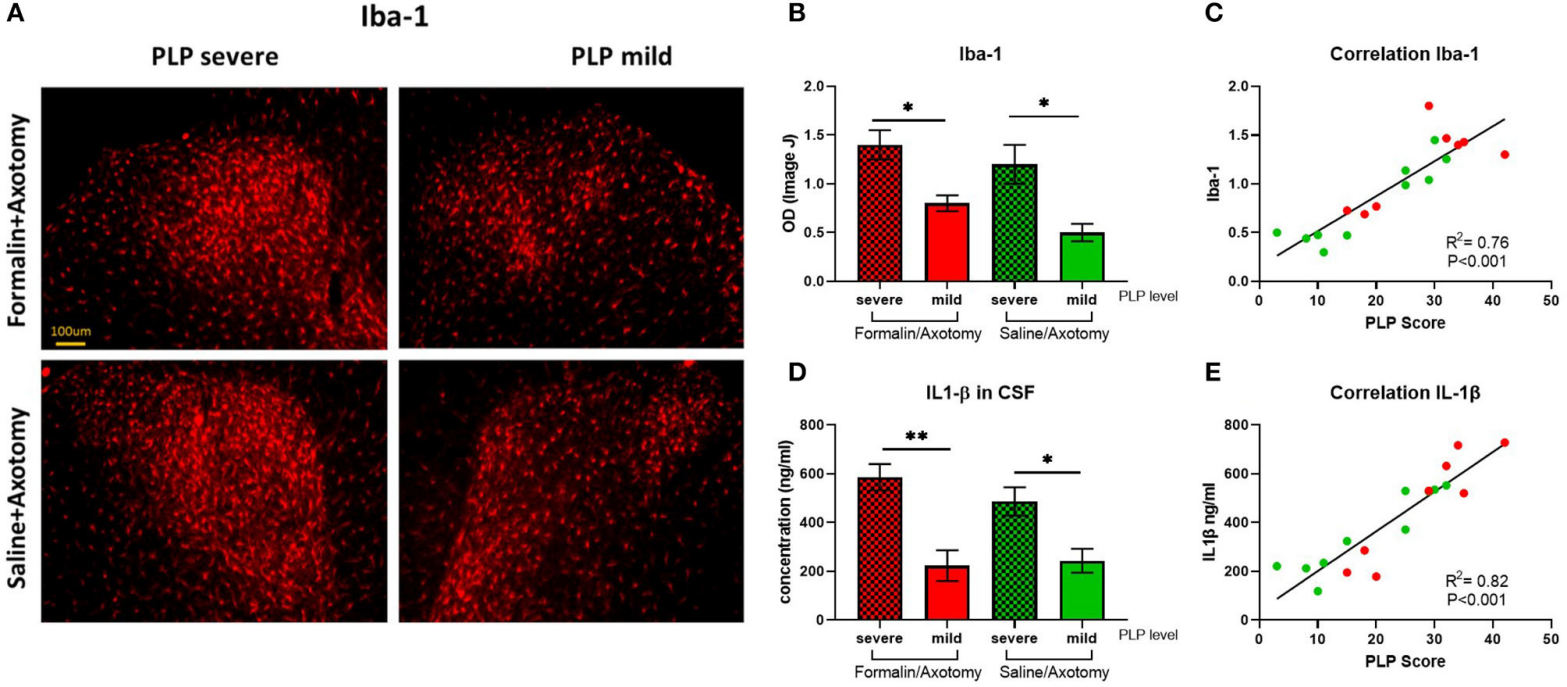
**(A)** Immunostaining of spinal dorsal horn for Iba-1 in animals with severe and mild PLP behavior. **(B)** Evaluation of Iba-1 immunodensity in the lumbar spinal cord. **(C)** Correlation analysis of Iba-1 immunodensity and PLP scores. **(D)** Evaluation of IL-1β levels in CSF of animals with severe and mild PLP behavior. **(E)** Correlation analysis of IL-1β levels and PLP scores. *P < 0.05, **P < 0.01 between the indicated groups.

Inflammatory marker Iba1 expressed by activated microglia was upregulated in animals with severe PLP behavior compared to animals with mild PLP symptoms in both formalin and saline groups (*P* < 0.05). Immunostaining showed dense upregulation in the medial part of the dorsal horn in animals with high PLP scores, with much sparser distribution in the mild PLP animals (Figures 10A,B). Correlation analysis showed positive correlation between the density of Iba-1 immunostaining and PLP scores (Figure 10C).

The level of inflammatory mediator IL-1β detected in the CSF by ELISA was upregulated in animals with severe PLP behavior compared with mild PLP scores (*P* < 0.01 and 0.05 for formalin- and saline-injected animals, respectively, Figure 10D). Again, the formalin-treated animals with high PLP scores appeared to show stronger upregulation than the saline cohorts, but this did not reach statistical significance, most likely due to the selection of the animals in both groups based on their PLP severity scores regardless of pretreatment. Correlation analysis showed a positive correlation between the level of IL-1β and PLP scores (Figure 10E).

#### GAD 65/67 and CGRP

**Figure 11:**
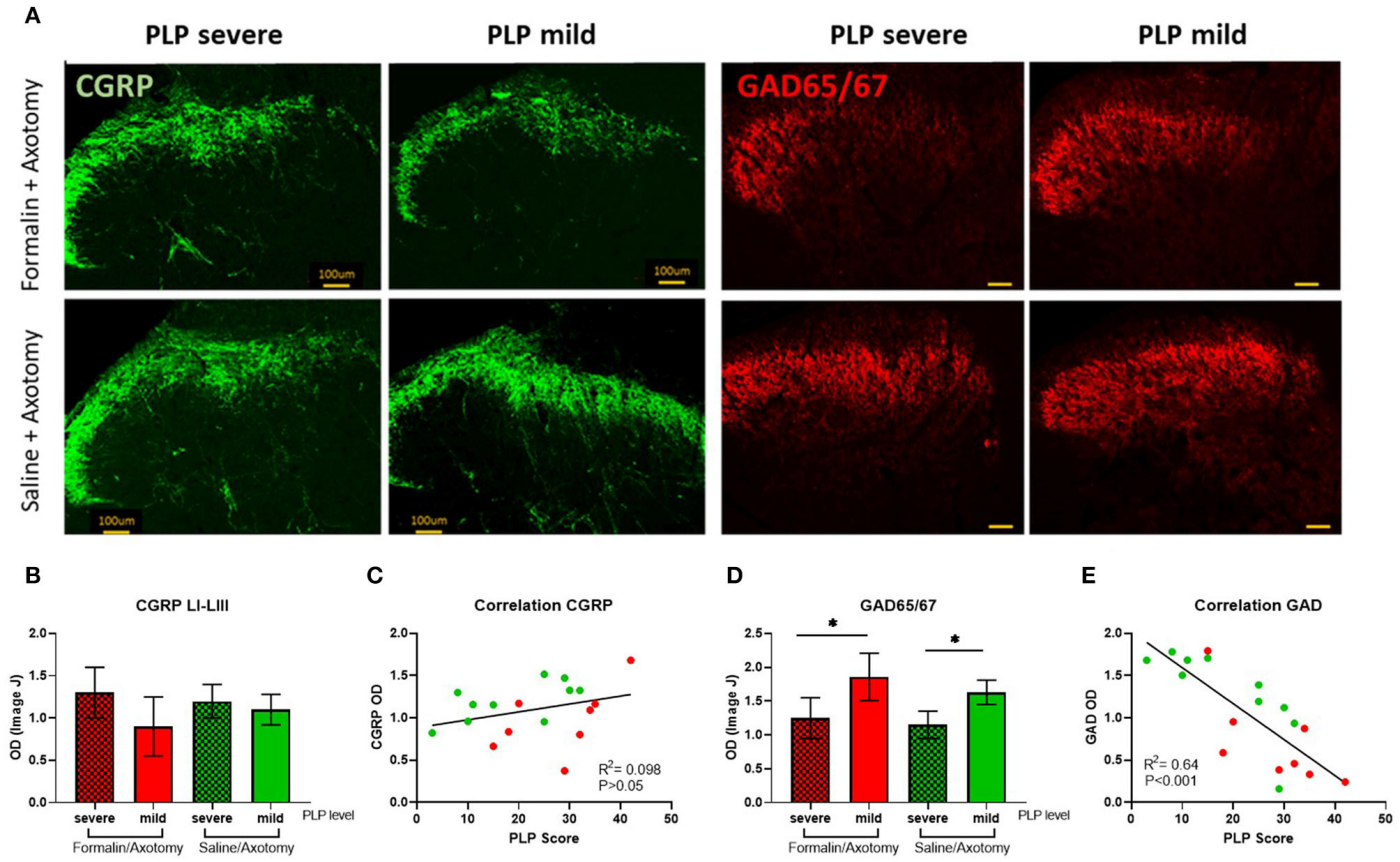
**(A)** Immunostaining of spinal dorsal horn for CGRP and GAD65/67 in animals with severe and mild PLP behavior. **(B)** Evaluation of CGRP immunodensity in the spinal laminae LI-III from lumbar levels. **(C)** Correlation analysis of CGRP Immunodensity and PLP scores. **(D)** Evaluation of GAD65/67 immunodensity in the lumbar spinal cord. **(E)** Correlation analysis of GAD65/67 immunodensity and PLP scores. *P < 0.05 between the indicated groups.

Expression of GAD65/67, an enzyme involved in the synthesis of the inhibitory neurotransmitter GABA, and CGRP as a marker of afferent nociceptive fibers were also analyzed in the spinal dorsal horn. The expression of CGRP was comparable between groups (Figures 11A,B). A slight upregulation of CGRP immunostaining was observed in the deeper dorsal horn laminae in animals with severe PLP that could indicate extended sprouting into deeper laminae (Figure 11A), but the overall CGRP expression was not statistically different regardless of pretreatment or PLP severity. Correlation analysis did not show a significant correlation between the density of CGRP immunostaining and PLP scores (Figure 11C).

GAD65/67 was downregulated in animals with severe PLP behavior after formalin or saline injection, compared to the animals with milder behavior (*P* < 0.05, Figures 11A,D). The GAD65/57 appeared most dramatically reduced in spinal cords of animals showing severe PLP behavior after formalin pretreatment, particularly in the medial dorsal horn. Correlation analysis showed a negative correlation between the density of GAD65/67 immunostaining and PLP scores (Figure 11E).

## Discussion

PLP-like behavior was induced in rats by axotomy of the sciatic/saphenous nerves, preceded by formalin injection or constriction of the nerve, to mimic clinical scenarios of peripheral tissue damage in complex injuries prior to medically indicated amputation. Most animals displayed some degree of autotomy behavior following axotomy that has been considered indicative of dysesthetic pain in animal models (13–18), and may be useful as an approximation of PLP sensations in humans. Injury prior to axotomy led to more severe behavior development compared to animals without preceding injury, both for formalin as an inflammatory injury and for CCI as a traumatic peripheral nerve injury. Several markers in the spinal cord and CSF showed differences between animals in relationship to PLP severity.

Clinical studies showed that the presence of pre-amputation pain increases the risk of developing PLP (22, 25, 30–32) and can be predictive of its quality and location (19–25). This is also suggested by previous preclinical findings in rats showing enhanced autotomy by an injury (e.g., hindpaw formalin injection) prior to nerve transection (33). These data suggest the involvement of a “pain memory” in the formation of delayed pain sensations. We have additionally evaluated this hypothesis in our study, including directing the pre-amputation injury to specific parts of the hind paw. The study was based on the original observation of Katz et al. (20), where the thermal injury was applied to the medial and lateral hindpaw and digits prior to complete nerve axotomy, showing autotomy preferentially localized to the thermal pre-injured regions. In the current study, we used a similar pre-injury location approach by formalin injection prior to axotomy for hindpaw peripheral neurogenic inflammatory pain (34–36) for more prolonged nociceptive stimulus. The inflammatory injection was targeted to either the medial or lateral plantar hind paw to determine the effect of pre-amputation pain location on subsequent directed post-amputation autotomy, in order to model and to test the clinical observation that prior injury/inflammation leads to exacerbation of PLP and perhaps localization, which could be indicative of “pain memory.” Results suggested a relationship between the hind paw areas affected by autotomy and the location of pre-axotomy formalin, with more lateral formalin injections resulting primarily in autotomy targeting to the two most lateral digits, while medial injection led to autotomy spread over a wider area with three to four digits involved. In addition, animals injected with formalin displayed more severe overall PLP scores and more rapid severe PLP development than animals injected with saline, although, some animals in both groups developed PLP-like behavior.

In order to mimic nerve injury-associated pain that often occurs with severe extremity injuries leading to medically indicated amputation, we used the CCI model (27, 37) as a pre-axotomy traumatic nerve injury (1 day or 4 weeks prior to axotomy). The decision to medically amputate following irreparable traumatic limb injuries can range from immediate (within 24 after trauma) to several weeks after trauma, when limb salvage is considered as infeasible due to injury severity and a decision to amputate is indicated (38). In the current study, the rationale for performing axotomy at 1 day and 4 weeks post-CCI was to determine whether early vs. delayed amputation may result in differences in the subsequent PLP severity, and possibly provide some additional clinical guidance with regard to post-injury amputation timing. Although, results suggested possible improved outcomes when axotomy is done following the initial acute injury, differences between the two time points were non-significant. Regardless of axotomy timing, animals with CCI showed a markedly enhanced autotomy response, both with regard to earlier onset and magnitude of severity compared to sham animals. Interestingly, as in the formalin responses, a similar, more laterally directed PLP autotomy behavior was also observed following CCI injury. The severity of autotomy directed to the lateral digits was particularly robust following CCI prior to transection. Again, there were a number of sham pre-injured animals that also displayed PLP autotomy. The digit distribution of autotomy in sham injured animals appeared more widespread rather than the more laterally directed autotomy following CCI. Together, both the formalin and CCI findings suggest the possibility of a central rearrangement of pain processing signals (at spinal cord level or higher) that might underlie the retention of memory for the prior pain exposure.

Although, the precise mechanisms of PLP development are unknown, PLP is considered a special form of neuropathic pain and shares some common features described for other types of neuropathic pain. The underlying mechanisms are thought to be a mix of changes from the level of peripheral nerves, to the spinal cord and supraspinal areas involved in pain processing. In the case of PLP, cortical involvement and the reorganization of neuronal wiring after deafferentation has been extensively studied (39–41). This may underlie a basis for the development of a pain memory, and could be interesting to compare using preclinical models such as described here, using various locations and severity of pre-axotomy insults.

A caveat using the nerve transection model for PLP is that clinical PLP from amputated limbs occurs in the absence of any innervation due to complete limb removal. The primary sensory innervation to the plantar hindpaw derives from the tibial branch of the sciatic nerve, with contributions to medial plantar and dorsal hindpaw from sural, peroneal, and saphenous nerves (42–44). Thus, the major sources of innervation to the hindpaw are severed in the current model. Nevertheless, it is still possible that there is some residual hindpaw innervation that could contribute to nerve-induced pain. For example, cutaneous nerves and blood supply stay mostly unaffected. The presence of some intact nerve fibers could interact with severed nerves undergoing Wallerian degeneration and become an additional source of inflammatory mediators and ectopic discharges that affect the development of autotomy (45–47).

At the level of the spinal cord, sprouting and hyperactivity of the injured primary afferents into the superficial and deep dorsal horn laminae and changes in the expression of the pain-related neurotransmitters have been attributed as possible mechanisms of PLP development (48–50). In this study, we have compared some of the pain-related markers between animals that have developed severe PLP behavior (score >25) with those that developed milder forms (<25) by the end of the experiment. Although, it was not possible to achieve an equal number of animals in these two groups, since most developed cutoff PLP scores requiring termination, we were able to detect some changes that might be considered as underlying causes or correlates of PLP development. More detailed analysis and correlation with levels of biomarkers at different time points post-injury in future studies may reveal more dynamic changes in these biomarkers with PLP development. Nevertheless, several of the selected biomarker changes showed potential relationships with the expression of more severe PLP behavior.

Voltage-gated sodium channels are important determinants of sensory neuron excitability (51–54). The changes in their expression or function significantly affect the ability of the nervous system to process nociceptive information. A wealth of data from studies involving various types of neuropathic pain confirmed the involvement of sodium channels in the development and maintenance of neuropathic pain (55, 56). In the current study, we analyzed the expression of NaV 1.7 in the lumbar spinal cord. Substantial changes were observed in the expression of NaV 1.7 with markedly enhanced expression in the superficial dorsal horn in animals exhibiting severe PLP behavior. These changes might contribute to neuronal hyperexcitability with signals originating from the severed sensory neurons contributing to the misinterpretation of pain originating from the missing limb.

Reduced spinal inhibition as indicated by reduced immunopositivity for GABAergic neurons or GAD65/67 enzyme, together with electrophysiological recordings, has been described in several models of neuropathic pain (37, 57–62). In the current study, we observed a marked reduction in GAD65/67 expression in the lumbar spinal dorsal horn of animals displaying higher PLP behavior. These changes might underlie disinhibition as one of the mechanisms for PLP development, contributing to neuronal hyperexcitability and central sensitization. CGRP expression in the lumbar superficial dorsal horn was also analyzed but no significant differences between animals with severe and mild PLP behavior were found.

Upregulation in activated glial cells is another factor thought to contribute to chronic neuropathic pain and PLP (63–66). Proinflammatory cytokines released by activated glial cells in the spinal cord are involved in the hyperexcitability and development of central neuropathic pain after spinal cord injury and peripheral nerve injury (67–74). Elevated level of IL-1β has also been reported in the CSF of chronic pain patients (75, 76). In the present study, upregulation of Iba1 in the spinal cord of animals displaying strong PLP behavior further supports the role of glial participation in PLP development and persistence. In line with this observation, upregulation in the inflammatory cytokine IL-1β was detected in the CSF of animals with severe PLP behavior compared to those with milder signs of PLP.

In conclusion, we have developed a promising model that mimics PLP of differing severity and time course and have described some of the neurochemical changes that are associated with the severity of PLP-like behavior. There has been a paucity of preclinical experimental work in this field, resulting in limited exploration and development of robust treatments for PLP. The observed elevated severity of PLP following a prior insult parallels clinical observation and emphasizes the need for pre-emptive attention to the increased potential for PLP development, particularly for medically indicated amputations, which are nearly always preceded by injury or disease in the affected extremities. The present study provides a base for future investigations into the mechanism and treatment interventions for managing PLP.

## Data Availability Statement

The original contributions presented in the study are included in the article/Supplementary Material, further inquiries can be directed to the corresponding author/s.

## Ethics Statement

The animal study was reviewed and approved by experimental procedures were reviewed and approved by the University of Miami Institutional Animal Care and Use Committee and the Department of Defense Animal Care and Use Review Office and followed the recommendations of the Guide for the Care and Use of Laboratory Animals (National Research Council).

## Author Contributions

SJ involved in study design, methodology, data analysis and interpretation, manuscript writing, project administration, and supervision. HM involved in study design, methodology, data acquisition and analysis, and manuscript draft. MH involved in methodology, data acquisition, and manuscript revisions. BS involved in methodology, data acquisition and interpretation, and manuscript draft and revisions. SG involved in data acquisition and analysis, data management, and manuscript draft. JS involved in study design, data interpretation, manuscript revisions, and supervision. All authors have seen and approved the final version of the manuscript.

## Conflict of Interest

The authors declare that the research was conducted in the absence of any commercial or financial relationships that could be construed as a potential conflict of interest.

## Supplementary Material

Prior to the axotomy, animals were tested for the presence of hypersensitivity to tactile and cold stimuli (Supplementary Figure 1). Animals in both CCI groups developed hypersensitivity; *P* < 0.01 and 0.001 for CCI 1d and CCI 4w, respectively, compared to pre-injury threshold for tactile stimuli (Supplementary Figure 1A) and *P* < 0.05 and 0.001 for CCI 1d and CCI 4w, respectively, compared to pre-injury threshold for cold stimuli (Supplementary Figure 1B). At 4 weeks, animals with CCI injury were more sensitive compared to the sham animals at the same time point (*P* < 0.05 for tactile test and *P* < 0.01 for cold test). No differences between pre- and post-injury were observed in sham groups (*P* > 0.05 for 1d and 4w for both tests).

Supplementary Figure 1 Development of **(A)** tactile and **(B)** cold hypersensitivity in animals after CCI injury (prior to axotomy). ^*^*P* < 0.05, ^**^*P* < 0.01, ^***^*P* < 0.001 for pre- vs. post-CCI; ^#^*P* < 0.05, ^##^*P* < 0.01 for CCI vs. sham.
