# Supplementary figures and images for: Development of a Phantom Limb Pain Model in Rats: Behavioral and Histochemical Evaluation
Source: Front Pain Res (Lausanne). 2021 Jun 21;2:675232. doi: 10.3389/fpain.2021.675232 (PMC8915728; doi:10.3389/fpain.2021.675232)

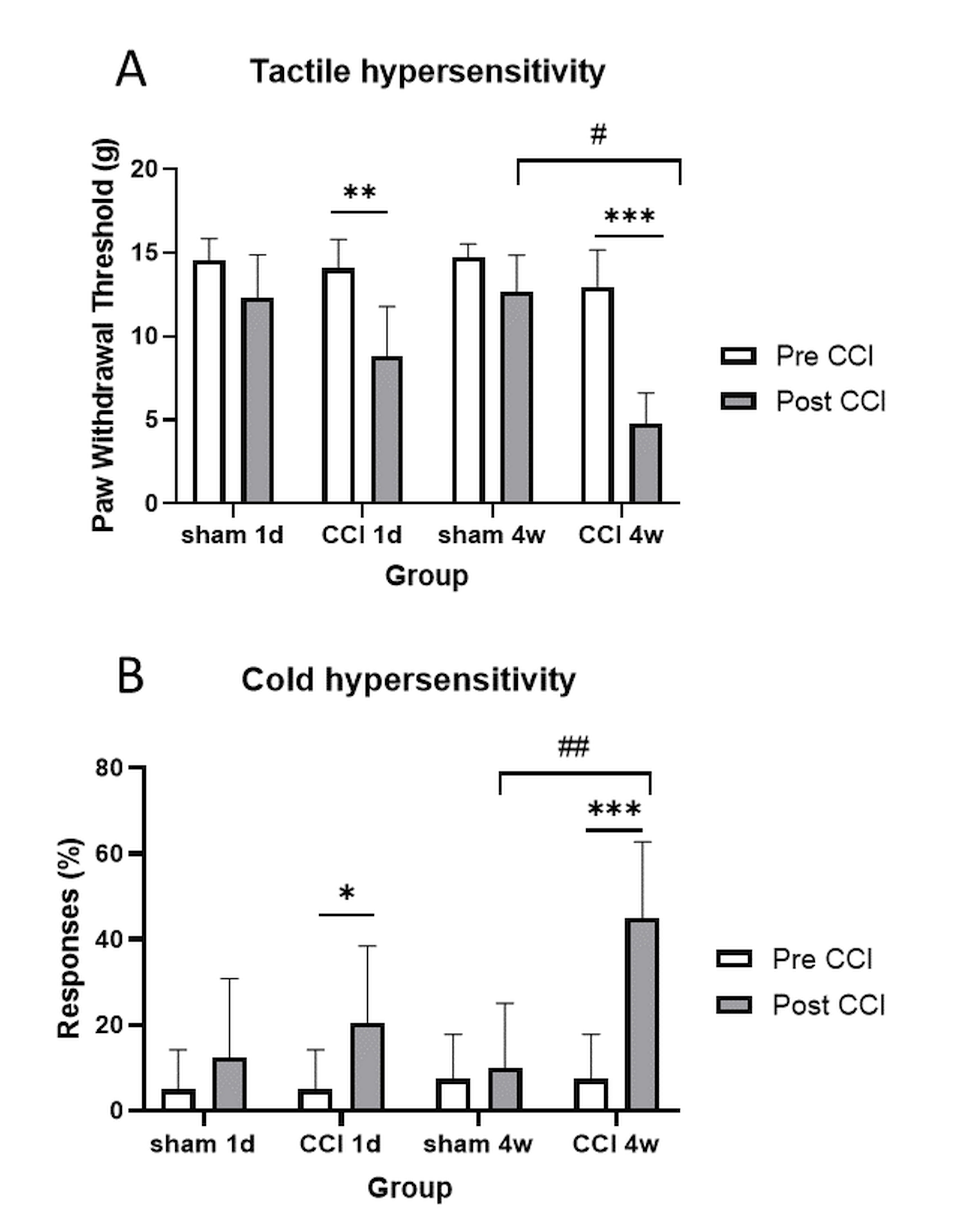

Supplement: Supplementary Figure 1 — Development of (A) tactile and (B) cold hypersensitivity in animals after CCI injury (prior to axotomy). *P < 0.05, **P < 0.01, ***P < 0.001 for pre- vs. post-CCI; #P < 0.05, ##P < 0.01 for CCI vs. sham. [file Image_1.JPEG]
